# Efficacy and safety of qiming granule combined with laser in the treatment of diabetic retinopathy: A protocol of randomized controlled trial

**DOI:** 10.1097/MD.0000000000025158

**Published:** 2021-03-26

**Authors:** Chuiren Zhou, Bi Long, Wei Huang, Ling Jiang

**Affiliations:** Chongqing Bishan District People's Hospital, Chongqing, China.

**Keywords:** diabetic retinopathy, panretinal photocoagulation, protocol, qiming granule, randomized controlled trial

## Abstract

**Background:**

Diabetic retinopathy is not only the most common complication of diabetes, but also 1 of the main causes of blindness, which seriously affects the physical and mental health of patients. Panretinal photocoagulation is a common method for the treatment of diabetic retinopathy, but it has some defects. Qiming granule has advantages in the treatment of diabetic retinopathy, but there is a lack of standard clinical research to verify it. Therefore, the purpose of this randomized controlled trial is to evaluate the efficacy and safety of qiming granule combined with laser in the treatment of diabetic retinopathy.

**Methods:**

This is a prospective randomized controlled trial to study the efficacy and safety of Qiming granule combined with laser in the treatment of diabetic retinopathy. Approved by the Clinical Research Society of our hospital. The patients are randomly divided into a treatment group (Qiming granule combined with laser treatment group) or control group (simple laser treatment group). The patients are followed up for 12 months after 6 months of treatment. Observation indexes include total effective rate, corrected visual acuity, macular fovea thickness, adverse reactions and so on. Data are analyzed using the statistical software package SPSS version 18.0 (Chicago, IL).

**Discussion:**

This study will evaluate the clinical efficacy and safety of qiming granule combined with laser in the treatment of diabetic retinopathy. The experimental results of this study will provide a reliable reference basis for clinical use of qiming granule combined with laser in the treatment of diabetic retinopathy.

**Trial registration:**

OSF Registration number: DOI 10.17605/OSF.IO/ZEQPB.

## 1 Introduction

With the change of diet, the aging of the population and the influence of the environment, the prevalence rate of diabetes has increased significantly. According to the statistics of the International Diabetes Federation, there are about 415 million people with diabetes in the world. It is estimated that the number of people with diabetes will reach 642 million by 2040.^[1]^ Diabetic retinopathy (DR) is a diabetic microvascular complication. As 1 of the main diseases causing blindness, diabetic retinopathy has become the main cause of visual disability and blindness in young adults.^[2]^ About 1/3 of diabetics will develop symptoms of diabetic retinopathy, and 1/3 of them will definitely have severe retinopathy or macular edema.^[3]^ In addition to the impact on vision, the existence of diabetic retinopathy also means an increased risk of systemic vascular complications, constantly endangering the lives of patients.^[2]^

Panretinal Photocoagulation (PRP) is a common method to treat and control the progression of DR.^[4,5]^ However, in addition to the therapeutic effect, PRP can also increase the temperature of local tissue of the eye, resulting in thermal degeneration of local tissue, destruction of blood-retinal barrier, macular edema.^[6]^ Damage to retinal ganglion cell axons, resulting in a continuous decrease of peri-optic disc nerve fibers, and complications such as vision loss and visual field defect caused by photocoagulation injury.^[7]^

Traditional Chinese medicine is an effective treatment of Chinese traditional medicine, which is widely used in the clinic. Qiming granule is a compound preparation of traditional Chinese medicine, which can improve the function of retinal microcirculation, reduce vascular permeability, reduce microvascular leakage and promote the absorption of macular edeman.^[8]^ There is evidence that qiming granule combined with PRP can reduce the expression of serum inflammatory factors, inhibit neovascularization, and help to improve the clinical efficacy of patients with DR.^[9]^ However, there is no clinical study on the efficacy and safety of qiming granule combined with PRP in the treatment of diabetic retinopathy. Therefore, we intend to use this randomized controlled trial (RCT) to evaluate the efficacy and safety of qiming granule combined with PRP in the treatment of diabetic retinopathy.

## 2 Materials and methods

### 2.1 Study design

**Figure 1:**
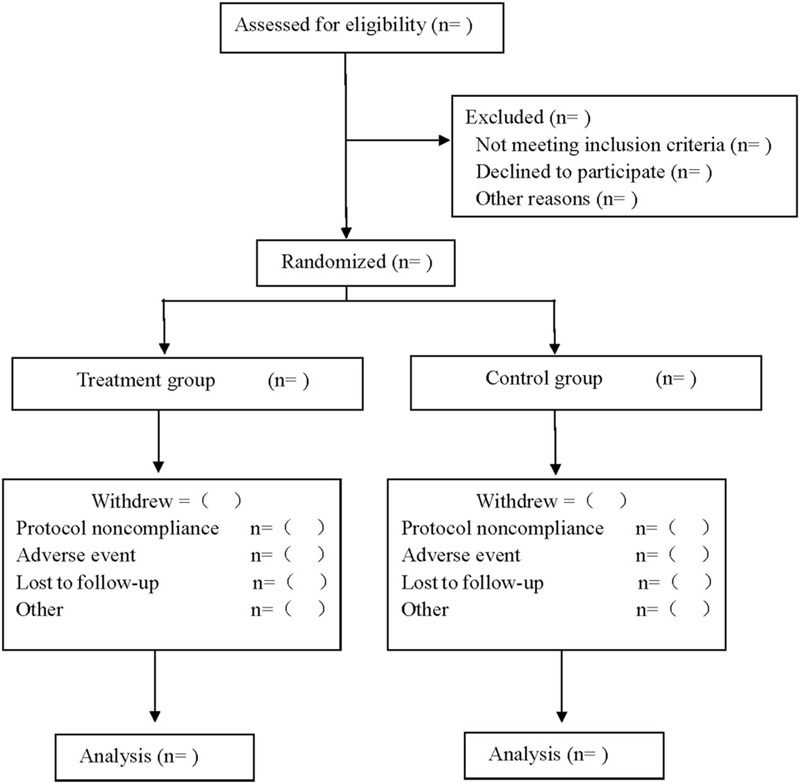
Flow diagram.

This is a prospective RCT to study the clinical efficacy and safety of qiming granule combined with laser in the treatment of diabetic retinopathy. This study will follow the comprehensive trial reporting standard.^[10]^ The flow chart is shown in Figure 1.

### 2.2 Ethics and registration

This research scheme is in line with the Helsinki Declaration and has been reviewed by the Clinical Research Ethics Committee of our hospital. This protocol is registered at OSF (registration number: DOI 10.17605/OSF.IO/ZEQPB). Before being randomly divided into groups, all patients are required to sign an informed consent form, and they can choose whether or not to continue the trial at any time.

### 2.3 Sample size

The calculation of sample size is based on the mean and standard deviation of best corrected visual aculty (BCVA) at 3 months after surgery. According to the results of the pilot study, the experimental group is 0.37 ± 0. 14, and the control group is 0.44 ± 0.13. Set α = 0.025, 1 sided test, β = 0.20. Calculated by the PASS15.0 software, each group needs 60 participants, with an estimated withdrawal rate of 10%, and each group will include 67 participants.

### 2.4 Patients

Inclusion criteria:① Diagnostic criteria and staging standards of DR: Adopted international disease severity scale for DR proposed by the Global Diabetic Retinopathy Project Group in 2002.^[11]^ The diagnosis is confirmed by fundus fluorescein angiography, and retinopathy stage II to IV; ② age range18 to 70 years; ③ blood glucose control 5–7.8 mmol / L; ④ signed informed consent.

Exclusion criteria: ① significantly impaired liver function, defined as ALT above 1.5 times upper limit of the normal level; ② patients who underwent other clinical trials in the last 3 months; ③ patients with poor glycemic control; ④ with severe primary diseases, such as cardiovascular, liver or kidney disease or mental illness; ⑤ accompanied by other ocular complications, such as glaucoma, severe cataracts, poor vision, retinal detachment, ophthalmic nerve disease, uveitis and retinopathy not related to DM.

### 2.5 Study design

Eligible participants are randomly assigned to either the treatment group or the control group in a 1:1 ratio using a random tool based on a central network. The random sequence randomization is performed without any stratification by independent statisticians who do not participate in the implementation of the experiment or statistical analysis using SAS 9.3 software (SAS Institute, Cary, NC). The clinical research coordinator enters participant information on the tablet and is given a random number. The research assistant gets the allocation of participants from the computer. Throughout the study, the research assistant is responsible for screening, recruiting participants, and assigning random numbers to participants who have been included. Results the evaluator is responsible for the evaluation of the scale. Considering the actual operational nature of acupuncture, participants and practitioners may be aware of random distribution. However, the evaluators of research results and the statisticians of data statistics and analysis are not informed of the distribution situation.

### 2.6 Intervention measures

All patients are given oral western medicine routine hypoglycemic drugs, and the medication is recorded.

In the control group, the laser treatment is carried out by the unified treatment team. After the compound tropicamide fully dilated pupil, obucaine hydrochloride eye drops are anesthetized. PRP is treated with 532 fundus laser therapeutic apparatus (Lumenis, Novus Spectra). Macular grid photocoagulation is performed first (spot diameter 100 μm, function 80–100mW, exposure time 0.1 s), followed by panretinal photocoagulation (posterior polar spot diameter 200 μm, peripheral spot diameter 300 to 500 μm, exposure time 0.15 to 0.20 s, total photocoagulation 1200–1600), 3 to 4 times to complete. The interval is 1 week at a time. Fundus fluorescein angiography is reexamined for 3 months to decide whether to supplement photocoagulation therapy.

On the basis of the control group, the observation group is given qiming granule (Zhejiang Wansheng, Chinese medicine Z20090036, Specification 4.5 g/ bags), taken with warm water, 1 bag per time, 3 times a day. The medicine is taken continuously for 6 months.

### 2.7 Evaluation criteria and efficacy judgment

(1) Total effective rate: refer to the clinical efficacy evaluation standard of Chinese Ophthalmology Clinical efficacy Diagnostic Standard.^[12]^ Obvious effect: Microaneurysms and other symptoms all or most disappeared, visual acuity improved more than 3 lines, visual field expanded 10 to 15 degrees or more; Effective: Microaneurysms and other symptoms significantly relieved, visual acuity improved more than 2 lines, visual field expanded 5 to 10 degrees or more; Ineffective: the above standard are not met or symptoms are aggravated. Total effective rate = (significant number + effective number)/total number∗ 100%.
(2) The BCVA and central macular thickness before and after treatment, BCVA examination will be performed using a standard logarithmic acuity chart, and the results are converted LogMAR recording method, and the central macular thickness is measured using optical coherence tomography.
(3) Adverse reactions: nausea, dizziness, rash, visual functional damage, and other discomforts occurred during the treatment.

### 2.8 Data collection and management

All patients are followed up for 12 months after treatment, and the visual acuity and curative effect are evaluated with the same equipment and method before treatment at 1 day, 1 months, 3 months, 6 months and 12 months after treatment. The whole process of data collection and recording is carried out by 1 or 2 assistants. Personal information about potential participants and registered participants will be collected, shared and stored in a separate storeroom to protect pre-trial, in-trial, and post-trial confidentiality. The access to the database will be restricted to the researchers in this study team.

### 2.9 Statistical analysis

The collected data are analyzed by SPSS 18.0 software. A chi-square test is used for counting data, mean ± standard deviation ($\bar{x}\pm s$) is used for measurement data, independent sample *t* test is used for normal distribution. Mann-Whitney *U* test is used for skewed distribution. When *P* *<* .05, and the difference is considered to be statistically significant.

## 3 Discussion

Diabetic retinopathy is an eye disease with specific changes in diabetes, and it is 1 of the most common and serious microvascular complications of diabetes.^[13]^ The main pathological changes in DR are ischemia and hypoxia in local retina, loss of fundus pericytes, disturbance of ocular vascular microcirculation, changes in the vascular wall and hemorheology, and finally induce neovascularization.^[3,14]^ At present, clinical treatment of DR mainly includes retinal laser photocoagulation, vitrectomy, anti-vascular endothelial growth factor drugs and so on, but the therapeutic effect is not ideal.^[2,15]^

Qiming granule is the first proprietary Chinese medicine approved by National Medical Products Administration for the treatment of DR. It is composed of Radix Astragali, Radix Puerariae, Radix Rehmanniae, Fructus Lycii, semen Cassiae, Fructus Rehmanniae, Radix Puhuang and Leech.^[16]^ Qiming granule has the effects of benefiting Qi, nourishing the liver and kidney, dredging collateral and clear eyes, enhancing immune function, promoting cell growth, improving microcirculation, lowering blood sugar and lipid. It has become 1 of the most widely used proprietary Chinese medicines in the treatment of DR in China.^[17]^ Animal experimental studies have found that qiming granule can reduce the level of blood glucose in rats, reduce the degree of retinal angiopathy, reduce the thickness of retinal capillary basement membrane,^[18]^ and inhibit retinal neovascularization by inhibiting inflammatory response and oxidative stress in DR rats.^[19]^

PRP is an effective method for the treatment of DR, which can reduce the release of neovascularization factors induced by ischemia and hypoxia and delay the progression of the disease.^[4]^ However, because PRP destroys the retinal photoreceptor retinal pigment epithelial complex, macular edema, visual function damage, visual field defect and other side effects will occur.^[5,20]^ There is evidence that qiming granule combined with fundus laser can promote macular edema absorption, reduce leakage, improve visual acuity and improve the total effective rate of treatment.^[21]^ Since there is no standard clinical study to evaluate the efficacy of qiming granule combined with laser in the treatment of diabetic retinopathy, we intend to evaluate its efficacy and safety through prospective RCTs.

This study also has some limitations: This study is a single-center randomized controlled study. The included population is regional, which may affect the results. At the same time, due to the influence of treatment, this study can not achieve strict double-blind, may have a certain impact on the results.

## Author contributions

**Data curation:** Chuiren Zhou, Bi Long.

**Formal analysis:** Chuiren Zhou.

**Funding acquisition:** Ling Jiang.

**Investigation:** Bi Long.

**Methodology:** Bi Long, Wei Huang.

**Resources:** Wei Huang.

**Software:** Wei Huang, Ling Jiang.

**Writing – original draft:** Chuiren Zhou, Bi Long.

**Writing – review & editing:** Chuiren Zhou, Ling Jiang.
